# Postpartum family planning attitudes among Turkish women: development of a reliable and valid scale

**DOI:** 10.1017/S1463423623000476

**Published:** 2023-10-18

**Authors:** Zeynep Sedef Varol, Meltem Çiçeklioğlu

**Affiliations:** 1 Department of Public Health, Division of Epidemiology, Faculty of Medicine, Dokuz Eylül University, Turkey; 2 Department of Public Health, Faculty of Medicine, Ege University, Turkey

**Keywords:** attitude, family planning, health belief model, postpartum, reliability, validity

## Abstract

**Aim:**

The aim of this study was to develop a scale based on the Health Belief Model (HBM) to assess the family planning (FP) attitudes of postpartum women with 0- to 12-month-old infants residing in eight neighbourhoods of the Bornova province, Izmir, Turkey.

**Introduction:**

Family planning is an integral component of maternal and infant health during the postpartum period and is a fundamental aspect of healthcare services in the prenatal and postnatal period.

**Methods:**

The Postpartum Family Planning Attitude Scale (PFPAS) was developed in four stages: item pool development, content validity evaluation, pilot study, and reliability and validity assessment. The PFPAS was administered to 292 women. The developed scale comprised 27 items and six sub-dimensions. Cronbach’s alpha coefficient was used to evaluate the reliability of the scale. Construct validity was evaluated using confirmatory factor analysis.

**Findings:**

Cronbach’s alpha coefficient was 0.88, indicating good reliability. Confirmatory factor analysis validated the structural validity of the scale, with a chi-square/degree of freedom ratio of 2.24, an RMSEA value of 0.068, and a CFI value of 0.95. The lowest and highest possible scores for the PFPAS were 27 and 135, respectively, with a mean total score of 105.32 ± 11.91.

## Background

Postpartum family planning (PPFP) is the prevention of unplanned pregnancy and closely spaced pregnancies during the first year after childbirth (WHO, 2013). Closely spaced pregnancies within the first year after delivery are the most dangerous for mother and child, increasing the risk of fatal outcomes such as infant and child fatalities, maternal mortality, and maternal morbidity, as well as perinatal health issues such as preterm, low birth weight, and small for gestational age (Conde-Agudelo *et al*., 2006; DaVanzo *et al.*, 2007; Rutstein, 2008).

Contraception has immediate health benefits because it prevents unplanned births, which reduces mother and newborn mortality and morbidity. To ensure optimal protection, a woman should begin using a FP technique as soon as the guidelines suggest (WHO, 2018). In order to provide proper FP knowledge and encourage early adoption of a FP strategy following a birth, FP counselling should essentially begin during antenatal care. Unfortunately, women prefer to use PPFP techniques only after they resume sexual activity or start their periods (Rossier and Hellen, 2014). Traditional and gendered norms for reproductive health services have an impact on healthcare access barriers (WHO, 2014).

Women’s willingness to engage in postpartum services should be viewed as an opportunity. Women are more willing to accept FP counselling during the prenatal, delivery, and postpartum periods because they have the most interaction with health staff and receive the most healthcare services. The attitudes that encourage or discourage women from using FP during the postpartum period must be identified so that the systems that supply and coordinate healthcare services related to PPFP can function more effectively and provide more effective counselling services (Erenel *et al.*, 2011).

It is crucial to use a scale appropriate for the social structure to evaluate the beliefs and attitudes that determine a person’s health behaviours when deciding on the subject matter and delivery strategy of the counselling service to be offered during the postpartum period (Jaccard *et al.*, 1996; Kongnyuy *et al.*, 2007; Arias *et al.*, 2018; Madrigal *et al.*, 2019). The Health Belief Model (HBM) was the first theory developed specifically to explain health-related behaviours (Janz and Becker, 1984). HBM was developed as a systematic approach to promoting public health through the recognition, explanation, and forecasting of preventive health behaviour. It is one of the most well-established and widely used health behaviour theories (Janz and Becker, 1984; Orji *et al.*, 2012).

The study’s goal is to develop a scale based on the HBM to assess the FP attitudes of postpartum women in Bornova, Izmir, with infants aged 0 to 12 months. Ege University Faculty of Medicine Clinical Research Ethics Committee decision No. 70198063-050.06.04 granted ethical approval.

## Methods

**Figure 1.**
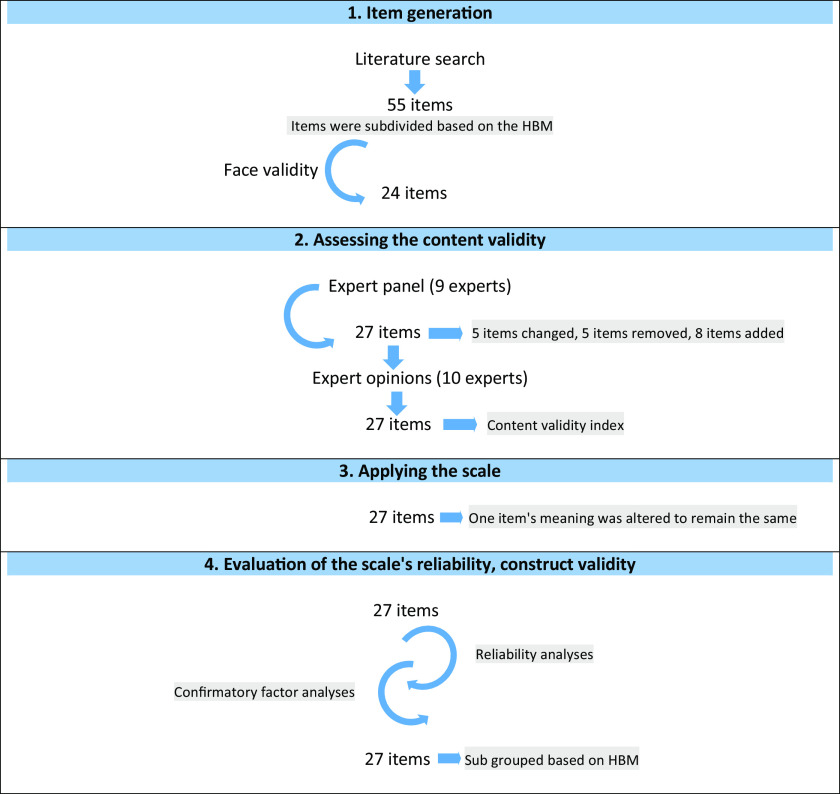
The process of developing the PPFP scale

It is a methodological study to develop a valid and reliable postpartum women’s FP attitude scale. The process of developing the scale was accomplished in the four stages as follows Figure 1.

### First step: item generation

The tools used in the literature to evaluate postpartum mothers’ attitudes towards FP and the efficacy of FP education initiatives were compiled. The data for the educational resources about FP and sexual health that were created using the HBM was gathered. A question pool of 55 questions was created from the collection of data on participants’ attitudes towards the items and FP approaches. The pool of 55 items was reduced to 24 items in the evaluation with the researcher and three public health specialists by combining or removing items in terms of meaning and content.

### Second step: assessing the content validity

In terms of HBM sub-dimensions and overall, an expert panel was formed to assess the scale’s suitability for the study’s goal. The panellists included the researcher, three public health specialists, two public health research assistants, a medical education expert, a midwifery department lecturer, and a midwifery department research assistant. The panel recommended that five of the items’ wording be changed, five items be removed because they contain repetitive claims, and eight elements related to perceived benefits and barriers be added. The scale was reorganized in accordance with the ideas, resulting in a scale of 27 items. Following the expert panel, ten people, including three public health experts, two public health nursing specialists, four midwifery lecturers, and one midwifery research assistant, assessed the 27-item scale for content validity. If the item is a candidate to explicitly measure the specified feature, ‘(a) Item represents the feature, appropriate’; if the item is on subject but needs to be edited or changed, ‘(b) The item needs some correction’ or ‘(c) The item needs a lot of correction’; if the item does not represent the specified feature, ‘(d) The item does not represent the feature, it is not suitable’. If the experts marked an item as ‘(b) or (c)’, they were asked to make suggestions for that item. Following expert advice, the responses and suggestions were analysed. After dividing the number of experts who marked the options ‘The item represents the feature, suitable’ and ‘The item needs some correction’ for each item by the total number of experts, the ‘content validity index’ (CVI) was calculated for each item. Subsequently, these indices were evaluated using the Davis technique^7^(Davis, 1992). The CVI of the scale was found to be 0.96, indicating that it is sufficient in terms of item content validity as it exceeds the threshold of 0.80. Following the CVI evaluation, the research group assessed the expert recommendations on seven items for easier comprehension, and sentence structures were rearranged.

### Third step: applying the scale

The finalized scale was applied to women living in eight socioeconomically disadvantaged neighbourhoods of Bornova, where the Bornova Municipality Health Affairs Directorate monitors pregnant women and babies via midwives. A sample size of at least 270 people, or ten times the number of scale items, was determined (Morgado *et al.*, 2017). The population consisted of 302 women who had registered with the Bornova Municipality Health Directorate with babies aged 0 to 12 months. The scale was administered to 292 women with children aged 0-12 months who agreed to participate in the study during the data collection process. Data were collected by visiting women’s homes and using face-to-face interview technique between September 7 and November 2, 2016.

### Fourth stage: evaluation of the reliability, validity, and scoring of the scale

Positive statements make up 16 of the scale items, while negative statements make up 11 of them. Negative statements (Items 1, 6 and 11-19) were reverse-coded to ensure consistency in the scale’s interpretation. The scale’s items were graded with Likert-type scoring ranging from 1 to 5 (strongly disagree, disagree, undecided, agree, and strongly agree) in the evaluation for negative statements, Likert scoring was coded as the inverse. The scale yielded the lowest possible value of 27, and the highest possible value was 135.

Cronbach’s alpha reliability coefficient was used to assess the reliability of the scale. The construct validity of the scale was determined using confirmatory factor analysis, and the compatibility of the scale’s sub-dimensions with the model was evaluated.

The Bartlett sphericity test *P*-value and the Kaiser-Meyer-Olkin (KMO) value were calculated.

Confirmatory factor analysis (CFA) is used to test the measurement model’s validity. This method employs a pre-built model to generate a latent variable (factor) from observed variables (Livote and Wyka, 2009). In this study, the factor structure representing six sub-dimensions was determined from the start using the HBM model.

Fit statistics were developed in order to evaluate the model’s goodness of fit (Morgado *et al.*, 2017). These statistics assess how well the designed model corresponds to reality, revealing the model’s structural validity. In CFA, chi-square/degrees of freedom statistics, comparative fit index (CFI), normed fit index (NFI), standardized root mean square residual (Standardized RMR), and root mean square error of approximation (RMSEA) were used to assess the fit of the model constructed on HBM.

LISREL 8.7 (LInear Structural RELations) and SPSS 26 software package were used to analyse the data. For all statistical analyses, the level of significance was set at *P* < 0.05, and the relationships were evaluated within the 95% confidence interval.

## Results

In the present study, the characteristics of the study participants were described within the primary healthcare setting. The average age of women was 27.90 ± 5.94, with 51.4% (*n* = 150) of them having completed primary education or higher. Among the women, 90.1% (n = 262) were homemakers and did not engage in income-generating work, and 19.9% (*n* = 58) did not have health insurance. Conversely, 60.3% of spouses had an education level beyond primary school, while 25.7% were employed in insecure and irregular jobs, and 19.5% did not have health insurance.

Table 1 presents the scores calculated for the scale items that were identified through the analysis of validity and reliability. Among them, Item 16, which pertains to fear of the side effects of contraception methods, has the lowest item mean score on the scale, with a value of 2.96 ± 1.04. On the other hand, Item 4, which relates to the belief of being able to get pregnant after unprotected sexual intercourse, has the highest item average score of 4.51 ± 0.57.

**Table 1.** Responses of women to the postpartum family planning attitude scale

Items*	1* N %	2* N %	3* N %	4* N %	5* N %	Mean ± SD
Perceived susceptibility						
Breastfeeding is a reliable form of contraception.	65 22.3%	126 43.2%	21 7.2%	77 26.4%	3 1.0%	3.59 ± 1.31
It is harmful to my health to become pregnant again within 2 years after giving birth.	1 0.3%	23 7.9%	21 7.2%	184 64.0%	60 20.5%	3.97 ± 0.79
If I become pregnant again within 2 years after giving birth, there is a risk that my newborn may be small, premature, and even die.	3 1.0%	34 11.6%	109 37.3%	121 41.4%	25 8.6%	3.45 ± 0.85
Perceived severity						
Possibility of pregnancy after unprotected sexual intercourse is a concern for me.	0 0%	2 0.7%	5 1.7%	128 43.8%	157 53.8%	4.51 ± 0.57
I do not consider withdrawal method to be an effective way of preventing pregnancy.	1 0.3%	22 7.5%	40 13.7%	132 45.2%	97 33.2%	4.03 ± 0.90
I have concerns about the potential infertility risks associated with contraceptive methods.	66 22.6%	141 48.3%	25 8.6%	56 19.2%	4 1.4%	3.72 ± 1.06
Perceived benefits						
Taking birth control pills reduces menstrual bleeding and regulates periods.	0 0%	33 11.3%	122 41.8%	86 29.5%	51 17.5%	3.53 ± 0.91
Using an intrauterine device (IUD) provides long-term protection against pregnancy.	1 0.3%	9 3.1%	32 11.0%	128 43.8%	122 41.8%	4.24 ± 0.79
Using condoms does not affect the quality of breast milk.	0 0%	1 0.3%	114 39.0%	91 31.2%	86 %29.5	3.90 ± 0.83
Using a contraceptive method ensures not getting pregnant within 2 years after giving birth.	0 0%	5 1.7%	16 5.5%	146 50.0%	125 42.8%	4.34 ± 0.66
Perceived barriers						
The cost of contraceptive methods is a significant barrier for me.	64 21.9%	179 61.3%	21 7.2%	27 9.2%	1 0.3%	3.95 ± 0.83
I am unaware of where I can obtain contraception for free.	147 50.3%	77 26.4%	5 1.7%	62 21.2%	1 0.3%	4.05 ± 1.18
It is challenging for me to access healthcare facilities where I can obtain contraception for free.	56 19.2%	214 73.3%	3 1.0%	18 6.2%	1 0.3%	4.05 ± 0.69
I am hesitant to discuss contraceptive methods with healthcare professionals, such as doctors, nurses, and midwives.	63 21.6%	186 63.7%	6 2.1%	37 12.7%	0 0%	3.94 ± 0.86
Contraceptive methods seem too complicated and difficult for me to use.	40 13.7%	172 58.9%	15 5.1%	64 21.9%	1 0.3%	3.64 ± 0.98
I am worried about the potential side effects of contraceptive methods.	16 5.5%	101 34.6%	32 11.0%	140 47.9%	3 1.0%	2.96 ± 1.04
Using contraception goes against my religious beliefs.	72 24.7%	180 61.6%	14 4.8%	26 8.9%	0 0%	4.02 ± 0.81
My partner does not want us to use contraception.	71 24.3%	169 57.9%	2 0.7%	46 15.8%	4 1.4%	3.88 ± 1.00
Cue to action						
It is my responsibility as a woman to prevent pregnancy.	32 11.0%	92 31.5%	9 3.1%	155 53.1%	4 1.4%	2.98 ± 1.16
My partner’s encouragement to use contraception has a positive effect on me.	1 0.3%	13 4.5%	12 4.1%	191 65.4%	75 25.7%	4.12 ± 0.70
Seeking advice on contraception from healthcare providers has a positive effect on me.	0 0%	3 1.0%	2 0.7%	147 50.3%	140 47.9%	4.45 ± 0.57
Knowing other family members or friends who use contraception encourages me to use it too.	0 0%	65 22.3%	6 2.1%	137 46.9%	84 28.8%	3.82 ± 1.08
Easy access to contraception has a positive effect on my willingness to use it.	1 0.3%	6 2.1%	6 2.1%	175 59.9%	104 35.6%	4.28 ± 0.64
Self-efficacy						
To prevent pregnancy, I am willing to go to a healthcare facility to obtain contraception method and service.	0 0%	10 3.4%	9 3.1%	119 40.8%	154 52.7%	4.43 ± 0.72
If we need to use a condom, my partner and I can correctly use it.	0 0%	58 19.9%	35 12.0%	110 37.7%	89 30.5%	3.79 ± 1.09
I can take the birth control pill regularly and without forgetting every day.	1 0.3%	78 26.7%	64 21.9%	85 29.1%	64 21.9%	3.46 ± 1.12
I can easily go to a healthcare facility to have an IUD inserted.	2 0.7%	20 6.8%	17 5.8%	119 40.8%	134 45.8%	4.24 ± 0.89

* 1 = strongly disagree, 2 = disagree, 3 = undecided, 4 = agree, 5 = strongly agree.

Table 2 presents the average scale scores based on the socio-demographic and economic status of the participants. Women with lower levels of education, women whose spouses have lower levels of education, women whose native language is Kurdish, women without health insurance coverage, women whose spouses lack health insurance coverage, and women whose spouses are employed in insecure or irregular jobs had significantly lower total scale scores.

**Table 2.** Scale score averages based on women’s socio-demographic and economic variables

Descriptive variables		*n*	Mean ± SD	*P*-value*
Age	25 and below	118	104.68 ± 11.53	0.450
	Above 25	174	105.75 ± 12.17	
Education	Primary school and below	142	102.82 ± 11.45	0.000
	Above primary school	150	107.68 ± 11.88	
Spouse’s education	Primary school and below	116	102.31 ± 12.25	0.000
	Above primary school	176	107.30 ± 11.27	
Native language	Turkish	187	106.89 ± 12.04	0.003
	Kurdish	105	102.52 ± 11.18	
Spouse’s social class	Insured – continuous	217	106.92 ± 11.56	0.000
	Uninsured – not continuously	75	100.68 ± 11.79	
Health insurance	Has health insurance	234	106.38 ± 11.78	0.002
	Does not have health insurance	58	101.03 ± 11.53	
Spouse’s health insurance	Has health insurance	235	106.25 ± 11.80	0.007
	Does not have health insurance	57	101.49 ± 11.65	
Total		292		

* The *P*-value was calculated for the independent samples t-test.

The reliability of the scale was evaluated using Cronbach’s alpha coefficient, which yielded a score of 0.878, indicating high reliability. A detailed analysis of Cronbach’s alpha coefficients for scale items is presented in Table 3. The sub-dimensions of the scale had the following Cronbach’s alpha values: Perceived Risk (0.457), Perceived Severity (0.500), Perceived Benefits (0.540), Perceived Barriers (0.763), Cues to Action (0.592), and Self-Efficacy (0.632). The low Cronbach’s alpha values for the sub-dimensions are attributable to the limited number of items in the sub-dimensions. Furthermore, the Kaiser-Meyer-Olkin (KMO) measure was excellent at 0.88, and Bartlett’s sphericity test *P*-value was significant (< 0.001), indicating sufficient correlation between scale items for factor analysis.

**Table 3.**
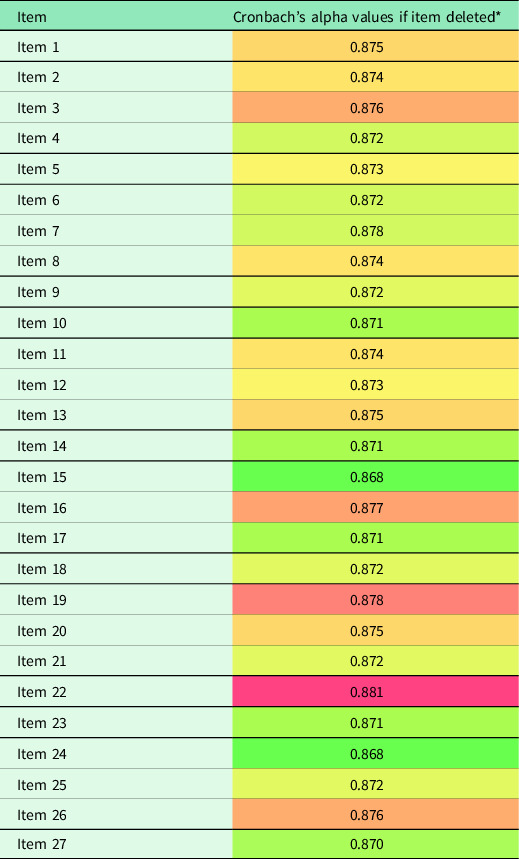
Cronbach’s alpha values of scale

*The harmonious values of colours span from red to green, with green exhibiting greater harmony and red exhibiting greater discordance.

The suitability of the structural model was tested using CFA due to the direct assignment of items to specific sub-dimensions in this study. The scale’s chi-square degrees of freedom, RMSEA, and NFI values were well-matched; the SRMR value was found to be acceptable. Although the scale’s fit indices do not fully fit, the majority of them are quite close to the good fit indices. Table 4 shows the summary fit values of the scale as a result of CFA.

**Table 4.** Model’s goodness of fit values

Fit indices	Good fit values*	Acceptable fit values*	Summary of fit values
*χ* ^2^/df	*χ* ^2^ /df < 2.5	*χ* ^2^/df < 5	2.240
RMSEA	RMSEA < 0.07	0.07 ≤ RMSA < 0.8	0.068
Standardized RMR	0.00 < SRMR < 0.05	SRMR < 0.10	0.065
NFI	NFI > 0.90	0.90 < NFI < 0.95	0.918
CFI	CFI > 0.92	0.90 < CFI < 0.95	0.953

* Fit indices and values of scale were given for the sample size is *n* > 250, and the number of observable variables is provided for the range of items between 12 and 30 (Byrne, 2016).

**Figure 2.**
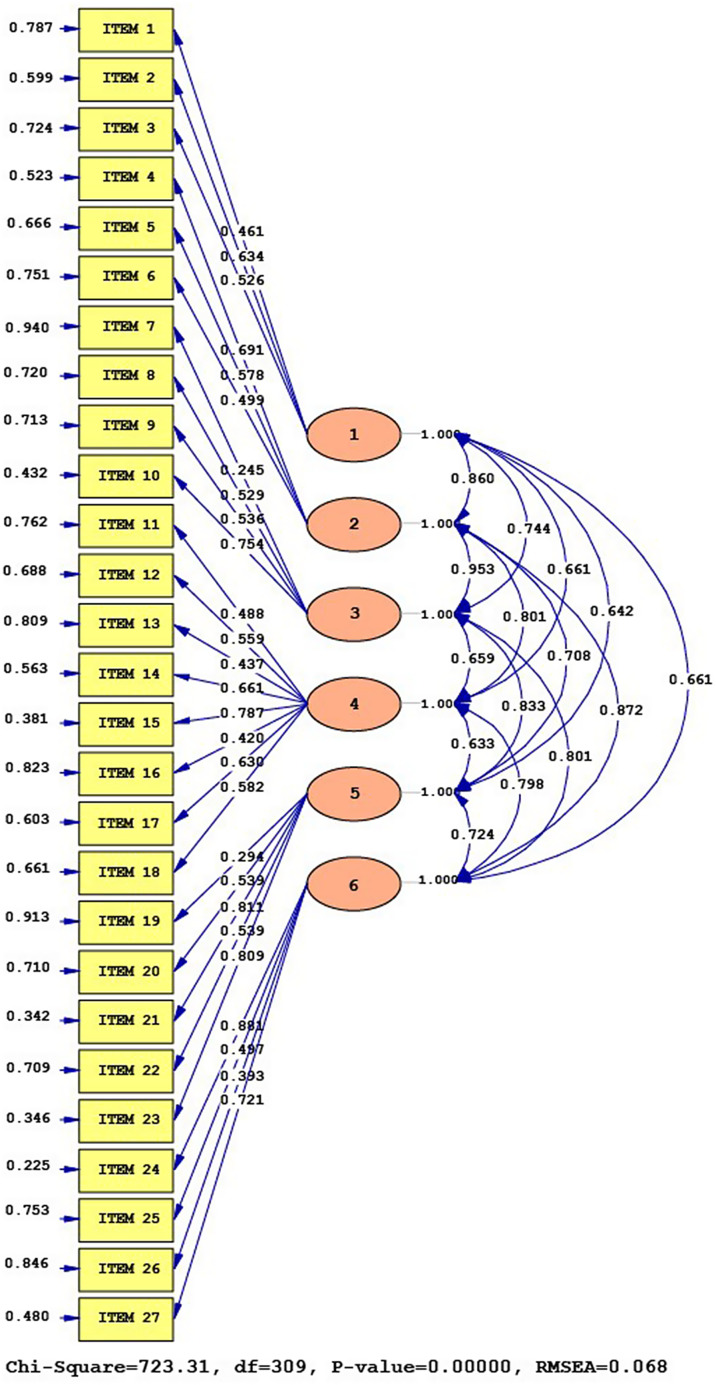
Structural Equation Model of PPFPS

The structural equation model analysis of the scale’s six-dimensional structure based on the HBM theoretical structure is illustrated in Figure 2. The scale correlation coefficients between the factor and the items vary between 0.25 and 0.88. Item 10 suppressed Item 7, which was categorized under the ‘perceived benefits’ factor. Similarly, Items 21 and 23 suppressed Item 19, which was under the ‘taking action’ factor, and Item 24 suppressed Item 26, which was under the ‘self-efficacy’ factor. The error variances range from 0.23 to 0.94, with Item 7 having a poor error variance of 0.94. Furthermore, Item 7 demonstrated a factor load distribution greater than 0.3 under three different factors.

## Discussion

This study aimed to develop and evaluate the Postpartum Family Planning Attitude Scale based on the Health Belief Model (HBM) to assess postpartum women’s FP attitudes. Although several studies have been conducted in the literature to evaluate postpartum women’s attitudes towards family planning (FP) using the Health Belief Model (HBM) conceptual framework (Eisen *et al.*, 1985; Jaccard *et al.*, 1996; Czuk, 1997; Dulli *et al.*, 2016), the psychometric approach employed in the development process of the scale in this study is distinct from those previous studies. The study tested the scale’s suitability using confirmatory factor analysis (CFA) and its validity.

In the context of family planning (FP), factors such as perceived risk, fear of pregnancy complications, and fear of bodily changes have been identified as important considerations (Jaccard *et al*., 2003; Adams *et al.*, 2008). In the CFA conducted to test the adequacy of the model structure for the three items comprising the perceived risk sub-dimension, the factor-item correlation coefficients and error variance values are within the appropriate range. The higher error variance of Item 1, which includes the statement ‘Breastfeeding is a reliable form of contraception’ compared to other items in this sub-dimension, may be associated with traditional misconceptions about the effectiveness of breastfeeding as a contraceptive method (Naçar *et al.*,^2003^).

The person’s perception of the seriousness of the unwanted pregnancy and its consequences that encourages them to have a positive attitude towards FP is referred to as perceived seriousness. Perceived risk and perceived seriousness constitute the two components of the Health Belief Model (HBM) that determine an individual’s perceived harm in a given situation. The literature, particularly in the field of FP, has emphasized the relationship of these components with taking action, perceived benefits, and mediator factors (Jaccard *et al.*,^1996^; Brown *et al*., 2011; Hall, 2012). In the CFA conducted for the perceived seriousness sub-dimension, the factor loading coefficients between the factors and items, as well as the error variance values of the items, are within the appropriate range and consistent with the model. There is no difference among items in terms of representing the sub-dimension.

Perceived benefits are linked to the effectiveness and advantages of using FP methods to prevent unwanted and unhealthy pregnancies, in the presence of perceived barriers (Jaccard *et al.*, 1996; Ieda and Sagbakken, 2012; Heinrich, 2014; Sileo, 2014). In the CFA conducted for the perceived benefits sub-dimension, except for the seventh item, the factor loading coefficients between the factors and items are consistent with the model. The error variance values of the items, except for the seventh item, are within an appropriate range as well. Although Item 7 represents the benefits of oral contraceptive (OC) use, it is suppressed by Item 10. This indicates that Item 7 less effectively represents the perceived benefits sub-dimension compared to other items. It is emphasized in the literature that a lack of knowledge about OCs triggers misconceptions (Rosenberg and Waugh 1998; Hall, 2012; Ieda and Sagbakken, 2012). Similarly, in this study, the lack of awareness among half of the study group regarding the potential benefits of OCs in relation to menstrual bleeding has caused the seventh item to be acceptable but incongruent in the CFA.

Traditional norms and sociocultural barriers, which encompass misconceptions about the side effects of modern family planning methods, have been identified as factors that detrimentally affect their utilization. (Hester and Macrina 1985; DeClerque *et al.*, 1986; Jaccard *et al.*, 1996; Rosenberg and Waugh 1998; Heinrich, 2014). In the perceived barriers sub-dimension, the item-factor correlations and error variances are consistent with the HBM conceptual framework. Item 15, which includes the statement ‘Contraceptive methods seem too complicated and difficult for me to use’, exhibits lower item-factor correlation and higher error variance compared to other items. This may be attributed to the fact that the items in this sub-dimension are related to economic constraints and traditional norms that hinder access to healthcare services, while Item 15 specifically questions one’s ability to use contraceptive methods.

The process of behaviour change in individuals, wherein they evaluate existing signs and clues from their attitudes, is referred to as taking action (Rosenberg and Waugh, 1998). All the factors that the woman interacts with socially provide cues and internal approval regarding which action is more appropriate and feasible for women (Hall, 2012). Taking action is the fifth factor of the scale model developed. In the CFA where the suitability of the scale’s model structure was tested, the factor-item correlation coefficients were found to be consistent with the model, with the exception of Item 19, which was at the limit value. When examining the error variance values of the items, Item 19 was also at the boundary value but within an acceptable range. In this sub-dimension of the scale, Item 19 was reverse-coded. It is worth considering that the development of contraceptive methods has primarily focused on women’s needs, resulting in limited options for men (Arias *et al.*, 2018). As a result, it is possible that participants perceived Item 19 outside of gender norms, which contradicts the primary questioning purpose of the study and may have influenced their responses towards FP techniques.

Self-efficacy is a complex construct influenced by multiple factors, including social, demographic, psychological, fertility, and access to health services, which together shape an individual’s perception of conception and birth control (Rimer and Glanz, 2005; Heinrich, 2014). In the confirmatory factor analysis conducted to test the scale’s model fit, it was found that the factor-item correlation coefficients of the self-efficacy factor were consistent with the model. The error variance values of the items were within an acceptable range. However, Item 26, which questions the ability to use oral contraceptives regularly, represents the self-efficacy sub-dimension to a lesser extent compared to other items. This can be attributed to the lack of information regarding the use of oral contraceptives. It is possible that the participants’ lack of awareness about the potential benefits of regular oral contraceptive use influenced their perception of self-efficacy (Hall, 2012; Şensoy, 2018). On the other hand, Item 24 has the lowest error variance and the highest factor-item correlation coefficient in this sub-dimension. This item focuses on accessing primary healthcare facilities, while the other items in the self-efficacy sub-dimension are related to the implementation of contraceptive methods.

### Limitations

While using the HBM as a foundation for developing the scale’s conceptual framework is empowering, it also has its limitations. The model has been criticized for placing too much emphasis on individual factors and attributing responsibility to individuals rather than considering socioeconomic and environmental factors (Thomas, 1995). Additionally, the model’s effectiveness in identifying the needs of individuals and specific groups is dependent on its relationship with social, political, and environmental factors. In this study, the data were analysed within the context of gender, and no specific discussion of HBM was made outside of this context. Therefore, the fact that the study was conducted within the HBM conceptual framework has generated a lively debate.

## Conclusion

The results of the study indicate that the newly developed scale is both valid and reliable, with satisfactory methodological outcomes. The scale was constructed based on the six sub-dimensions of the Health Belief Model (HBM), and their validity and reliability were tested. This study is particularly significant because there is currently no specific scale in Turkey that focuses on regulating fertility in the postpartum period within the framework of health behaviour theory.

## References

[1] Adams A , Buckingham CD , Lindenmeyer A and Arber S (2008) The influence of patient and doctor gender on diagnosing coronary heart disease. Sociology of Health and Illness 30, 1–18. doi: 10.1111/j.1467-9566.2007.01025.x. 18254830

[2] Arias MLF , Champion JD , Soto NES , Navarro VN and Caudillo Ortega L (2018) Adaptation of the contraceptive behavior scale for Mexican heterosexual populations. Hispanic Health Care International 16, 56–61. doi: 10.1177/1540415318776445. 29781292

[3] Brown W , Ottney A and Nguyen S (2011) Breaking the barrier: the Health Belief Model and patient perceptions regarding contraception. Contracept 83, 453–458. doi: 10.1016/j.contraception.2010.09.010. 21477689

[36] Byrne, BM (2016). Structural Equation Modelling with AMOS: Basic Concepts, Applications, and Programming (3rd ed.). New York: Routledge.

[4] Conde-Agudelo A , Rosas-Bermudez A and Kafury-Goeta AC (2006) Birth spacing and risk of adverse perinatal outcomes. JAMA 295, 1809–1823. doi: 10.1001/jama.295.15.1809. 16622143

[5] Czuk CL (1997) Women’s Perceptions of Postpartum Appointment Keeping Barriers. Doctoral dissertation, Grand Valley State University, Allendale Charter Township, MI.

[6] DaVanzo J , Hale L , Razzaque A and Rahman M (2007) Effects of interpregnancy interval and outcome of the preceding pregnancy on pregnancy outcomes in Matlab, Bangladesh. BJOG: An International Journal of Obstetrics and Gynaecology 114, 1079–1087. doi: 10.1111/j.1471-0528.2007.01338.x. 17617195 PMC2366022

[7] Davis LL (1992) Instrument review: getting the most from a panel of experts. Applied Nursing Research 5, 194–197. doi: 10.1016/S0897-1897(05)80008-4.

[8] DeClerque J , Ong Tsui A , Abul-Ata MF , Abul-Ata MF and Barcelona D (1986) Rumor, misinformation and oral contraceptive use in Egypt. Social Science Medicine 23, 83–92. doi: 10.1016/0277-9536(86)90327-8. 3749967

[9] Dulli L , Eichleay M , Rademacher K , Sortijas S and Nsengiyumva T (2016) Meeting postpartum women’s family planning needs through integrated family planning and immunization services: results of a cluster-randomized in Rwanda. Global Health: Science and Practice 4, 73–86. 27016545 10.9745/GHSP-D-15-00291 PMC4807750

[10] Eisen M , Zellman GL and McAlister AL (1985) A health belief model approach to adolescents’ fertility control: some pilot program findings. Health Education & Behavior 12, 185–210. doi: 10.1177/109019818501200205. 3997538

[11] Erenel AŞ , Kavlak T and Bingöl B (2011) Kadınların Doğum Sonrası Altı Ay Sonunda Aile Planlaması Yöntemi Kullanma Durumu. Van Tıp Dergisi 18, 68–76.

[12] Hall KS (2012) The health belief model can guide modern contraceptive behavior research and practice. Journal of Midwifery Women’s Health 57, 74–81. doi: 10.1111/j.1542-2011.2011.00110.x. PMC3790325 22251916

[13] Heinrich RL (2014) Handbook of behavioral medicine. The American Journal of Psychiatry 143, 381–382. doi: 10.1176/ajp.143.3.381.

[14] Hester NR and Macrina DM (1985) The health belief model and the contraceptive behavior of college women: implications for health education. Journal of American College Health 33, 245–252. doi: 10.1080/07448481.1985.9935034. 4045018

[31] Ieda A and Sagbakken M (2012) Perceptions and Behaviour Related to Family Planning in a Rural Area in the Oromia region, Ethiopia Thesis Submitted as Part of the Master of Philosophy Degree in International Community Health., (June), 1–104. Retrieved 1 February 2023.

[15] Jaccard J , Dodge T and Dittus P (2003) Do adolescents want to avoid pregnancy? Attitudes toward pregnancy as predictors of pregnancy. *The Journal of Adolescent Health: Official Publication of the Society for Adolescent Medicine* 33, 79–83. doi: 10.1016/S1054-139X(03)00134-4. 12890598

[16] Jaccard J , Helbig DW , Wan CK , Gutman MA and Kritz-Silverstein DC (1996) The prediction of accurate contraceptive use from attitudes and knowledge. Health Education & Behavior 23, 17–33. doi: 10.1177/109019819602300102. 8822399

[17] Janz NK and Becker MH (1984) The health belief model: a decade later. Health Education Quarterly 11, 1–47. doi: 10.1177/109019818401100101. 6392204

[18] Kongnyuy EJ , Ngassa P , Fomulu N , Wiysonge CS , Kouam L and Doh AS (2007) A survey of knowledge, attitudes and practice of emergency contraception among university students in Cameroon. BMC Emergency Medicine 7, 1–7. doi: 10.1186/1471-227X-7-7. 17634106 PMC1933435

[19] Livote EE and Wyka KE (2009) Introduction to structural equation modeling using SPSS and AMOS. Structural Equation Model: A Multidisciplinary Journal 16, 556–560. doi: 10.1080/10705510903008345

[20] Madrigal JM , Atluri M , Radeke EK and Patel A (2019) Looking through the lens of a family planner to prioritize reproductive health among women with cancer. JCO Oncology Practice 15, e141–e152. doi: 10.1200/jop.18.00429. 30763204

[21] Morgado FFR , Meireles JFF , Neves CM , Amaral ACS and Ferreira MEC (2017) Scale development: ten main limitations and recommendations to improve future research practices. Psicologia: Reflexao e Critica 30, 1–20. doi: 10.1186/s41155-016-0057-1. PMC6966966 32025957

[22] Naçar M , Öztürk A and Öztürk Y (2003) The effect of family planning education given during postpartum period on the use of contraceptive methods. Journal of Clinical Practice and Research 25, 122–130.

[26] Orji R , Vassileva J and Mandryk R (2012) Towards an effective health interventions design: an extension of the health belief model. Online Journal Public Health Inform 4. doi: 10.5210/ojphi.v4i3.4321. PMC3615835 23569653

[25] Rimer BK and Glanz K (2005) Theory at a Glance A Guide For Health Promotion Practice, 2nd edition. Bethesda, MD: US Department of Health and Human Services, National Institutes of Health, National Cancer Institute.

[27] Rosenberg MJ and Waugh MS (1998) Oral contraceptive discontinuation: a prospective evaluation of frequency and reasons. American Journal of Obstetrics and Gynecology 179, 577–582. 9757954 10.1016/s0002-9378(98)70047-x

[28] Rossier C and Hellen J (2014) Traditional birthspacing practices and uptake of family planning during the postpartum period in ouagadougou: qualitative results. International Perspectives on Sexual and Reproductive Health 40, 87–94. doi: 10.1363/4008714. 25051580

[29] Rutstein SO (2008) Further Evidence of the Effects of Preceding Birth Intervals on Neonatal, Infant, and Under-Five-Years Mortality and Nutritional Status in Developing Countries: Evidence from the Demographic and Health Surveys. Working Papers No. 41. Calverton, MD: Macro International. https://dhsprogram.com/publications/publication-wp41-working-papers.cfm 10.1016/j.ijgo.2004.11.012 15820369

[30] Sileo KM (2014) Determinants of Family Planning Service Uptake and Use of Contraceptives among Postpartum Women in Rural Uganda. Retrieved 4 February 2023 from 10.1007/s00038-015-0683-x PMC4644123 25967466

[23] Şensoy N , Korkut Y , Akturan S , Tuncel B and Tuz C (2018) Factors Affecting the Attitudes of Women Toward Family Planning. Family Planning, Londrina: IntechOpen, pp. 33–50.

[32] Thomas LW (1995) A critical feminist perspective of the health belief model: Implications for nursing theory, research, practice, and education. Journal of Professional Nursing 11, 246–252. 7665800 10.1016/s8755-7223(95)80027-1

[33] WHO (2013) Programming Strategies for Postpartum Family Planning. Retrieved 27 January 2023 from https://www.who.int/publications/i/item/9789241506496

[34] WHO (2014) Ensuring Human Rights in the Provision of Contraceptive Information and Services - Guidance and Recommendations. Retrieved 29 January 2023 from https://www.who.int/publications/i/item/9789241506748 24696891

[35] WHO (2018) World Health Organization Department of Reproductive Health and Research (WHO/RHR) and Johns Hopkins Bloomberg School of Public Health/Center for Communication Programs (CCP), Knowledge for Health Project. Family Planning: A Global Handbook for Providers (2018 update). Baltimore and Geneva: CCP and WHO, 2018. Retrieved 30 January 2023 from https://www.who.int/publications/i/item/9780999203705

